# Creating and administering video vignettes for a study examining the communication of diagnostic uncertainty: methodological insights to improve accessibility for researchers and participants

**DOI:** 10.1186/s12874-023-02072-7

**Published:** 2023-12-15

**Authors:** Caitríona Cox, Thea Hatfield, Jordan Moxey, Zoë Fritz

**Affiliations:** The Healthcare Improvement Studies (THIS) Institute, University of Cambridge, Cambridge, United Kingdom

**Keywords:** Video-vignette, Analogue patients, Methodology, Diagnostic uncertainty

## Abstract

**Background:**

Studying clinician-patient communication can be challenging, particularly when research seeks to explore cause-and-effect relationships. Video vignettes – hypothetical yet realistic scenarios – offer advantages to traditional observational approaches by enabling standardisation and manipulation of a clinician-patient encounter for assessment by participants. While published guidelines outline stages to create valid video vignette studies, constructing high quality vignettes which are accessible to a wide range of participants and feasible to produce within time and budget restraints remains challenging. Here, we outline our methods in creating valid video vignettes to study the communication of diagnostic uncertainty. We aim to provide practically useful recommendations for future researchers, and to prompt further reflection on accessibility issues in video vignette methodology.

**Methods:**

We produced four video vignettes for use in an online study examining the communication of diagnostic uncertainty. We followed established guidelines for vignette production, with specific consideration of how these might be applied pragmatically to save time and resources. Scripts were pilot-tested with 15 laypeople, and videos with 14 laypeople; pilot-testing involved both quantitative and qualitative analysis.

**Results and discussion:**

We demonstrate the usefulness of existing guidelines, while also determining that vignette production need not necessarily be expensive or time-consuming to be valid. Our vignettes were filmed using an iPhone camera, and featured a physician rather than a professional actor; nonetheless, pilot-testing found them to be internally and externally valid for experimental use. We thus propose that if care is taken in initial script development and if pragmatic choices are made regarding filming techniques and pilot-testing, researchers can produce valid vignettes within reasonable time and budget restraints. We also suggest that existing research fails to critically examine the potential benefits and harms of online video vignette methodology, and propose that further research should consider how it can be adapted to be inclusive of those from underserved backgrounds.

**Conclusions:**

Researchers creating video vignette studies can adapt the video vignette development process to suit time and budget constraints, and to make best use of available technology. Online methods may be harnessed to increase participant accessibility, but future research should explore more inclusive vignette design.

**Supplementary Information:**

The online version contains supplementary material available at 10.1186/s12874-023-02072-7.

## Introduction

Various approaches exist for the study of doctor-patient communication [1, 2]. Observational studies of real doctor-patient interactions are not always feasible. Observing sensitive or emotive communication may be logistically and ethically challenging [3]. Moreover, observations do not allow for controlled manipulation of variables: they can explore correlations between communication behaviours and different outcomes measures, but they rarely explore causation [2, 4].

Vignette studies provide a useful alternative. A vignette is a “*short, carefully constructed description of a person, object, or situation, representing a systematic combination of characteristics*” [5]. Hypothetical yet realistic scenarios are shown to participants, who are then invited to respond [6]. Responses reveal participants’ beliefs, attitudes, judgments, knowledge, or intended behaviours with respect to the vignette context [5]. In experimental vignette studies, controlled modification of key variables (while keeping the remaining content of the vignettes constant) enables researchers to infer causal relationships [7, 8]. Manipulating one aspect of communication in isolation allows for greater standardisation compared with observational studies of real consultations [7, 9].

‘Analogue patients’ (APs) are often used in healthcare communication studies. APs watch or read vignettes depicting an interaction with a healthcare professional, and imagine themselves in the position of the patient [10–14]. Vignette studies using APs can also be helpful in overcoming ceiling effects, which occur in studies using real patients who are unwilling to criticise their own doctors [9, 15]. This may be particularly important when emotive measures (e.g. trust) are being examined – social desirability effects might result in real patients feeling pressured to give their own doctors higher values, positively skewing results.

The vignettes themselves can be presented using a range of modalities: written text (a narrative or a script), cartoons, pictures or videos [5, 7]. Written vignettes have been used to study doctor-patient communication [10, 16], but have been criticised for potential low external validity [17]. Video vignettes may facilitate better participant engagement, and increasingly have been used to study health communication [3]. Creating valid video vignettes is not, however, a straightforward process: many diverse factors must be considered, from developing verbal manipulations in a script to determining which camera angles to use.

Until the last decade, there was little evidence-based guidance or practical instruction for researchers developing their own video vignettes; even recently published vignette studies often fail to clearly report various methodological stages [3, 6, 7]. To provide practical guidance for researchers, Hillen et al. published recommendations on how to create valid video vignettes [3]. They suggested five phases: deciding if video vignettes are appropriate; developing a script; developing valid manipulations; converting the script to video and finally administering the videos. Other papers describe in detail the development of video vignettes, in healthcare research [18–22] as well as other areas [23–25].

Although such publications have provided researchers with guidance, creating and implementing video vignettes can still be a “*daunting task*”, not least due to the cost and logistics involved in producing realistic videos [3]. Additionally, researchers planning video vignette studies must consider diversity and inclusion. Increasingly, online methods are recognised as helpful in delivering video vignette studies. If carefully designed, they present an opportunity to increase accessibility for participants from underrepresented groups, but if not, they risk being exclusive and results under-representative. Ultimately, online video vignettes are a potentially valuable method for studying doctor-patient communication, but only if they are accessible to both researchers and to participants from a range of backgrounds.

Here, we outline our application of Hillen’s guidance to create video vignettes for a study examining the communication of diagnostic uncertainty. We detail our methodology with the intention of helping other researchers develop video vignettes. In reflecting upon our methodological choices, we provide insights into how video vignettes studies can be more accessible: both to researchers (by demonstrating that high-quality video vignettes can be produced within resource-limited environments), and to participants (by suggest ways in which the online delivery of vignettes can be adapted to be more inclusive to those from underserved groups).

## Study context and aims

### Background and wider research programme

Uncertainty is inherent to medicine – particularly in the diagnostic process [26–28] – yet issues surrounding the communication of diagnostic uncertainty to patients remain relatively underexplored. Although the GMC recommends that doctors explain to patients when they are uncertain about a diagnosis, they do not provide detail on how it might be done [29]. The study described here is part of a wider multidisciplinary programme of research, examining the practical, legal and ethical issues surrounding how diagnoses are formed, communicated and recorded.

As part of this research, we initially conducted two systematic reviews examining the communication of diagnostic uncertainty in primary care [30] and acute secondary care [31]. These demonstrated that research is limited by a lack of consensus on how diagnostic uncertainty is defined or measured, and found evidence for variation in how diagnostic uncertainty is communicated to patients in practice.

**Fig. 1:**
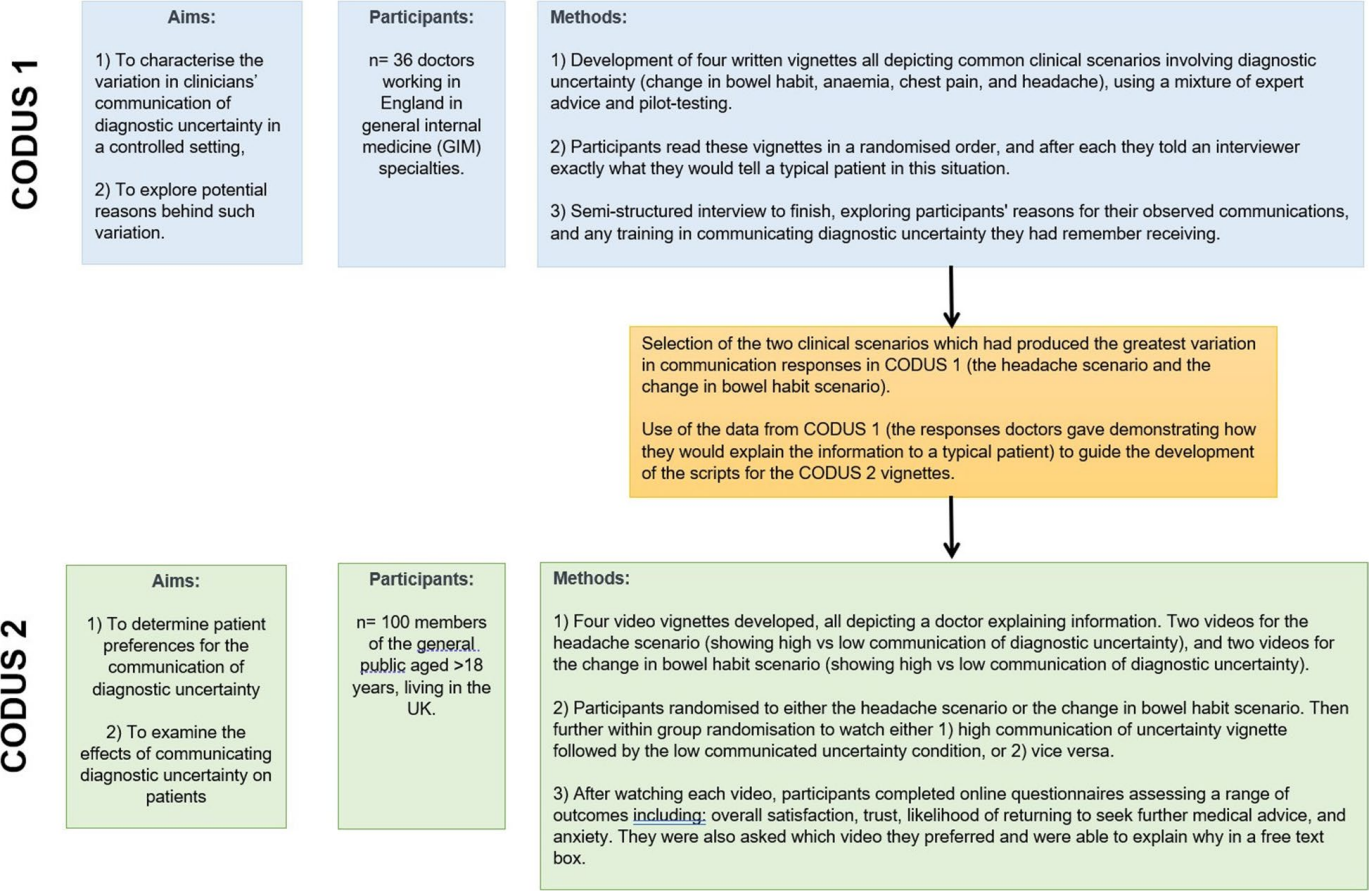
Overview of CODUS 1 and CODUS 2 studies

The vignette element of this research (Communication Of Diagnostic Uncertainty Study [CODUS]) involved two stages: 1) initial study involving doctors using written vignettes (CODUS 1), and 2) video vignette study involving patients (CODUS 2). Figure 1 provides an overview of these.

The methods and results of CODUS 1 are detailed elsewhere [32]. In this first study, we found significant variation in the communication of diagnostic uncertainty: some doctors went into detail about the uncertainty surrounding the diagnosis, while others did not explicitly acknowledge uncertainty at all. Participants described various and often conflicting justifications for their behaviours. Notably, we found doctors had differing opinions on the impact that communicating diagnostic uncertainty might have on their patients: some felt it might have a negative impact on the therapeutic relationship or patient anxiety, while others felt the reverse.

### Rationale behind the current video vignette study (CODUS 2)

**Fig. 2:**
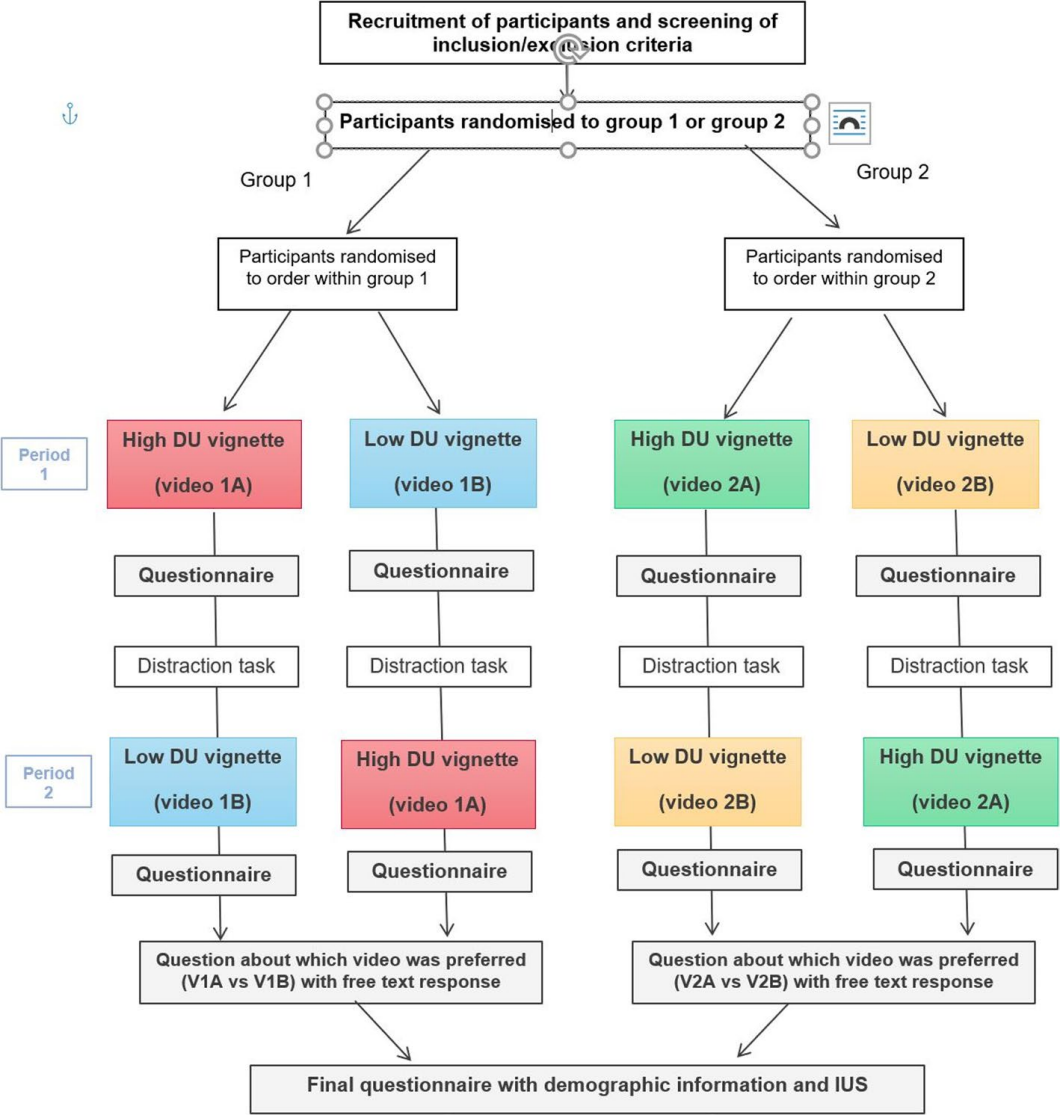
CODUS 2 study design

In light of these results, a second study (CODUS 2) aimed to examine the effects on patients of communicating diagnostic uncertainty in two varying clinical scenarios (see Fig. 2 for study design). For this, we developed four video vignettes (two depicting a headache scenario, and two for a change in bowel habit scenario). For each scenario, we developed one vignette depicting high communicated diagnostic uncertainty, and one depicting low communicated diagnostic uncertainty. All other aspects of communication were kept constant between the different conditions – the study aimed to isolate the communication of diagnostic uncertainty and investigate its impact on patients.

## Methods and pilot-testing results

**Fig. 3:**
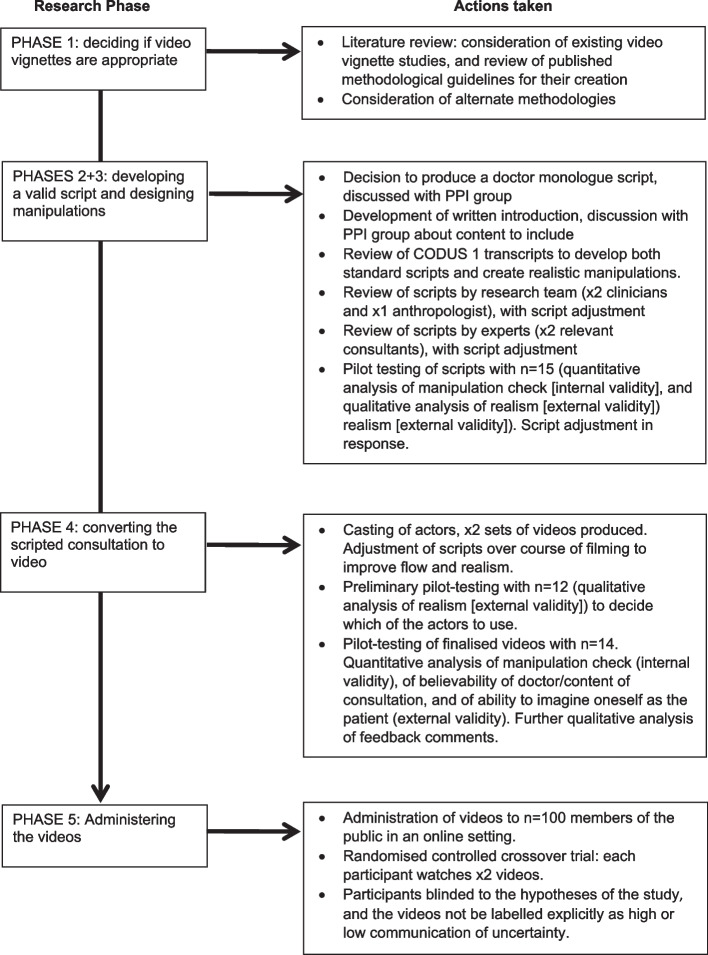
Phases of creating video vignettes and actions taken in our study (adapted from van Vliet et al (2013)) [18]

Here we outline our methods in developing the video vignettes, with emphasis on the steps we took in producing high quality vignettes despite time and budget restraints. Figure 3 provides an overview of the actions we took in developing the video vignettes against the research phases proposed by Hillen et al. [3] The development of the scripts and the pilot testing took place from February 2022 to October 2022; the main study data collection took place from December 2022 to March 2023.^1^

### Stage 1: deciding if using video vignettes is appropriate

In observational studies of real consultations, specific communication behaviours cannot be isolated and manipulated. In contrast, vignette methodology permits controlled manipulation of certain elements (such as the degree to which diagnostic uncertainty is communicated). As such, for our research questions, vignette methodology offers an advantage over observational studies of real consultations.^2^

The use of APs avoids ethical issues which might be associated with using real patients [3]. There is a theoretical concern – with some limited supporting evidence – that communicating uncertainty might have a negative impact on patient trust, satisfaction and perception of doctor competence [10, 33–35]. Using APs allows manipulation of the communication of diagnostic uncertainty without harming real patients.

Vignette methodology has some limitations. Vignettes may never be identical to real consultations: although APs can effectively put themselves in the position of the patient in the vignettes [9, 36], this is unlikely to be completely equivalent to a real patient responding to the communication. Doctors often tailor their communication to the specific patient – in vignette studies there can be no such adaptation of communication content, and nor can there be any discussion between the AP and the doctor. 

Considering these strengths and limitations, we concluded that vignettes would be the optimal methodology for addressing our research questions.

### Stages 2 and 3: developing a script with valid manipulations

We developed four scripts: high and low communicated diagnostic uncertainty for both the ‘change in bowel habit’ vignette (V1A and V1B), and for the ‘headache’ vignette (V2A and V2B) (see Table 1).

**Table 1:** Initial scripts

Script	Conditions
V1A	Change in bowel habit, high communication of diagnostic uncertainty
V1B	Change in bowel habit, low communication of diagnostic uncertainty
V2A	Headache, high communication of diagnostic uncertainty
V2B	Headache, low communication of diagnostic uncertainty

#### Doctor monologue vs. conversation

Existing studies vary in the use of doctor monologue [10] vs. scripted doctor-patient conversation [18, 19, 37]. Including both patient and doctor may create a more naturalistic vignette, but may negatively impact the external validity (as APs may find it more difficult to realistically imagine themselves in the role of the patient). Some studies indicate that this is more challenging if the vignette depicts a patient with distracting different characteristics to themselves (e.g. different age or gender) [3, 17]. This is theoretically grounded in the similarity-identification hypothesis: the notion that identification is increased by similarity between audience members and characters [38].

Although this idea is intuitively compelling, it currently lacks conclusive empirical support: “*the empirical evidence regarding the similarity hypothesis is mixed and combined with the strong theoretical and intuitive appeal of this hypothesis a more definitive investigation is needed*” [38]. In a systematic review on narratives used to convey health messages, a few studies reported a higher persuasiveness when characters in the narrative were similar to those watching it, but most found no differences [39]. A more recent study examining the effect of gender in vignette studies found no effect of gender congruence on self-reported video engagement [40].

This choice was discussed at two PPI group meetings; participants felt using a doctor only monologue might make it easier for participants to imagine themselves in the patient’s position.

#### Developing an introduction 

Vignette studies often contain an introduction to familiarise participants and provide background information. It may be written, an audio voiceover, or a video sequence using an actor introducing themselves as the patient [41].

A study comparing the use of a written vs. an audiovisual introduction demonstrated greater cardiovascular response when watching the latter, but did not find any differences in self-reported engagement or in perceived realism [41]. This study also assessed the impact of showing participants a conversation between a doctor and a patient, vs. a doctor monolog. Notably, participants who had an audiovisual introduction *and* who watched the doctor monologue version of the vignette had a lower emotional engagement than with the written introduction. The authors concluded that “*researchers who do not want to show the patient at all during the video-vignette consultations should consider using a written introduction*” [41].

We therefore developed written introductions. We used lay terms to increase comprehensibility, and included background information including symptoms experienced, patient location, and a clear timeline.

#### Standard script development

We developed a standard script for each scenario, using common elements from CODUS 1 transcripts to enhance ecological validity. Most responses followed a similar structure: initial introduction and reassessment of the clinical situation, explanation of the investigation results, discussion about the likely diagnosis with a suggested plan, and safety-netting. Two researchers – CC (a doctor working in internal medicine) and TH (an anthropologist) read though the transcripts and noted common phrases, such as “*your investigations are very reassuring*”, to use verbatim in the CODUS 2 standard scripts. We scripted the whole vignette, leaving no space for ad-libbed portions (to ensure that the only difference between the conditions would be the communication of diagnostic uncertainty).

#### Developing manipulations

The standard scripts were then manipulated to create high vs low communicated uncertainty conditions.

Drawing directly from CODUS 1 transcripts, we created a table with quotations demonstrating high vs low communicated diagnostic uncertainty. Again, by using examples of what doctors had actually said, we aimed to make the scripts as ecologically valid as possible. As the communication of diagnostic uncertainty is not a simple construct, we chose to vary multiple verbal segments. Our ‘high uncertainty’ scripts accumulated verbal segments from multiple doctors. We discussed and iterated these scripts, aiming to balance realism with manipulation success: we aimed to make manipulations which were distinguishable, without descending into caricature [3, 42].

The resulting high communicated uncertainty scripts were longer because of increased discussion. Length discrepancies have been noted in other vignette studies [18, 19]. Following others, we decided not to correct for length differences because they reflect real consultations. Explicitly explaining diagnostic uncertainty would likely take more time than not disclosing it, so keeping the length discrepancy is more realistic [43].

To isolate the impact of delivering information content about diagnostic uncertainty to patients, we only manipulated verbal elements; non-verbal communication (e.g. eye contact, body position, expressions) were kept as similar as possible between the videos. We note that it is impossible to entirely separate non-verbal and verbal communication – for example, the tone in which information is imparted and the speaker’s body language will naturally be somewhat influenced by the information itself [3]. Therefore, we emulated Gehenne et al.’s approach, aiming to keep the non-verbal behaviour broadly similar between different vignettes, but congruent with the content of the consultation [19].^3^

#### Refining scripts using expert opinion

Consulting relevant experts can help to establish realism at the script development phase [3]. We shared our scripts with a consultant gastroenterologist and consultant neurologist respectively. Small changes were made in response to their feedback – for example, the wording of V1B script was altered to include terms the gastroenterology consultant commonly uses in explaining an IBS diagnosis. Both experts felt that the scripts were medically accurate and believable.

#### Pilot-testing scripts

Video vignette studies frequently use pilot-testing but vary in the extensiveness of the process [19, 44]. Of note, published guidelines do not stipulate how many pilot participants are required. Our approach balanced the usefulness of feedback with the potential logistical challenges of repeated or extensive pilot-testing.

We undertook pilot-testing with a convenience sample of 15 participants who met the inclusion/exclusion criteria for the main study (mean age 34.8 years, range 19–67 years). They were laypeople without medical expertise, and were given all the scripts to read in a randomised order. Script-pilot testing focused on testing internal validity: were the manipulations sufficiently distinct in their communication of diagnostic uncertainty?

The communication of diagnostic uncertainty is a complex construct [45], which lacks a universal definition or validated tools for its measurement [46]. We were thus unable to replicate other vignette studies which have used validated multi-item instruments to test internal validity, as no such instruments exist for the construct (the communication of diagnostic uncertainty) [19]. Instead, we emulated a study which examined the communication of prognostic uncertainty, which used a single item to test manipulation sucess [47]. We asked participants how explicit the discussion of uncertainty surrounding the diagnosis was, using an 11-point scale (from 0 “not at all” to 10 “very”). For both the sets of scripts, the high uncertainty communication scripts were perceived as displaying significantly greater explicit communication of diagnostic uncertainty (*p* < 0.001) (Table 2).

**Table 2:** Script pilot-testing (two-tailed Wilcoxon Signed-Rank test, 0.05 significance level)

Pilot Participant number	Demographic data (gender, age)	How explicitly did the doctor discuss any uncertainty surrounding the diagnosis? 0(not at all)—10 (very explicitly)			
		**V1A**	**V1B**	**V2A**	**V2B**
**1**	F, 24	7	2	9	2
**2**	M, 26	9	3	8	1
**3**	F, 22	9	4	6	3
**4**	F, 59	10	8	10	7
**5**	M, 27	10	6	9	5
**6**	F, 25	9	6	8	4
**7**	F, 19	3	2	3	1
**8**	M, 25	10	1	9	1
**9**	F, 35	9	7	9	5
**10**	M, 55	8	4	8	4
**11**	F, 25	9	3	9	2
**12**	M, 63	7	4	8	2
**13**	F, 67	7	5	8	3
**14**	F, 21	8	3	7	4
**15**	F, 29	9	2	9	2
**Mean**		8.23	4.23	8	3.08
**SD**		1.8	2	1.7	1.8
***p*-value**		< 0.001		< 0.001	

Additionally, we garnered general feedback on the scripts, including comments on realism and understandability. Small changes were made in response, for example to reduce jargon.

### Stage 4: Converting the scripted consultations to video

Filming video vignettes can be prohibitively expensive and time-consuming, particularly if using professional film crews or consulting script advisors. Moreover, these costs can rapidly increase if pilot-testing of videos produces unsatisfactory results, necessitating amendments and re-shooting. Our decisions regarding filming and pilot-testing the videos were shaped by these realities.

#### Filming the vignettes: camera angle and production

Various camera angles have been used in existing video vignettes studies, for example showing only what the patient sees (facing the doctor), or alternating between the patient and the doctor [3]. Some studies have suggested that it is preferable to use alternate camera angles to increase perceived realism and emotional engagement [19, 41].

Since the COVID-19 pandemic, there has been a significant increase in telemedicine, including the use of video consultations [48]. We chose to use a doctor-only camera angle, (the doctor addressed the camera as though it is a patient), to replicate these increasingly common video consultations. As participants would likely complete the study online using their own devices, we hoped that the similarity to a video consultation would enable them to better imagine themselves in the position of a patient.

We filmed the vignettes using an iPhone, a tripod and a teleprompter application. Multiple takes of each vignette version were filmed. We chose not to pilot-test different takes due to time constraints – we instead selected the best take for each vignette condition and used these in pilot-testing (see below). These were chosen based on global assessments by the author team, taking into account the overall quality, naturalism of the delivery and consistency of affect and tone across the different vignette conditions.

Each vignette was filmed as one continuous take to avoid any transitions between clips which may have been distracting. This approach was more appropriate within our resource constraints as we did not contract a professional recording/editing team.

#### Use of actors

Some existing video vignette studies have used actors, while others have used real clinicians. As Hillen et al. discuss, there are potential advantages and disadvantaged to both: real clinicians may be more naturalistic and adept with medical terminology, while actors may be more comfortable in front of camera and better able to deliver consistency in style [3].

Based upon a previous vignette study in which pilot participants found an actor more realistic [18], we initially chose to employ a professional actor to play the doctor. The actor had previously worked with medical students in communication skills teaching. All four vignettes were filmed over the course of one day. Small amendments to make the script flow more naturally were made in response to feedback from the actor.

Unfortunately, early informal pilot-testing with a convenience sample of 10 laypeople suggested that the realism of the videos produced from this first day of filming was inadequate. These pilot-testers were shown the vignettes and asked for general feedback on their realism: they reported the actor to be unnatural in their tone and non-verbal communication. We subsequently reshot the vignettes using a medical doctor. We showed videos to the same sample of pilot-testers (without disclosing that this was a real doctor rather than an actor), and again asked them for general feedback on realism. We particularly asked them to compare the re-shot vignettes with the original versions. They universally preferred the videos with the doctor, describing them as more realistic. Subsequently we proceeded with these in the formal pilot-testing phase (see below).

As in our experience, amending scripts and reshooting videos in response to feedback can be essential to vignette validity. We urge researchers to account for possible reshooting costs when planning studies to avoid having to decide between compromised validity and excessive costs.

#### Pilot-testing videos

We pilot-tested videos with a convenience sample of 14 laypeople who met the proposed inclusion/exclusion criteria for the main study (mean age 34.9, age range 19–69). To replicate the conditions of the main study, each participant was shown the introductory text, before watching either videos V1A and V1B **or** V2A and V2B. Each pilot tester thus watched 2 videos, randomised to watch the A or B video first.

We used simple numerical scales to assess both internal and external validity, which have been used in pilot-testing in previous healthcare communication video vignette studies [49].

1. Internal validity testing (manipulation check): Participants rated on a 11-point scale how explicitly the doctor discussed any uncertainty surrounding the diagnosis, from 0 “not at all” to 10 “very explicitly”. For both sets of videos, the high uncertainty communication scripts were perceived as displaying significantly greater explicit communication of diagnostic uncertainty (*p* < 0.05) (Table 3).
2. External validity testing (realism): Participants rated on a 11-point scale how believable the doctor was, and how believable the content was, from “not at all” to “very”. They also rated on a 11-point scale to what extent they were able to imagine themselves in the position of the patients, ranging from “not at all” to “very strongly”. Participants rated the realism and their ability to imagine themselves as the patient highly (Table 4). Qualitative feedback was positive: participants praised the realism of the doctor character and the quality of the recordings.

**Table 3:** Video pilot-testing internal validity (two-tailed Wilcoxon Signed-Rank test, 0.05 significance level)

Pilot Participant number	Demographic data (gender, age)	How explicitly did the doctor discuss any uncertainty surrounding the diagnosis? 0(not at all)—10 (very explicitly)			
		**V1A**	**V1B**	**V2A**	**V2B**
**1**	M, 26	10	5	-	-
**2**	F, 29	8	4	-	-
**3**	F, 59	10	0	-	-
**4**	F, 31	10	0	-	-
**5**	M, 32	8	1	-	-
**6**	M, 23	10	6	-	-
**7**	F, 24	10	7	-	-
**8**	F, 27	-	-	9	4
**9**	F, 19	-	-	9	2
**10**	M, 30	-	-	9	7
**11**	F, 26	-	-	9	0
**12**	M, 30	-	-	7	1
**13**	M, 63	-	-	9	2
**14**	F, 69	-	-	6	4
**Mean**		9.4	3.3	8.3	2.9
**SD**		1	2.9	1.3	2.3
***p*-value**		< 0.05		< 0.05

**Table 4:** Video pilot-testing external validity testing

Pilot Participant number	Vignettes watched	Demographic data (gender, age)	How believable do you think the doctor was?	How believable do you think the consultation content was?	To what extent were you able to imagine yourself in the position of the patient ?
			**0 (not at all)- 10 (very)**	**0 (not at all)- 10 (very)**	**0 (not at all)- 10 (very)**
**1**	V2A and V2B	M, 26	9	10	10
**2**	V2A and V2B	F, 29	8	9	7
**3**	V2A and V2B	F, 59	10	10	10
**4**	V2A and V2B	F, 31	9	9	8
**5**	V2A and V2B	M, 32	8	8	7
**6**	V2A and V2B	M, 23	7	9	8
**7**	V2A and V2B	F, 24	8	9	8
**8**	V1A and V1B	F, 27	8	7	9
**9**	V1A and V1B	F, 19	9	6	6
**10**	V1A and V1B	M, 30	7	6	7
**11**	V1A and V1B	F, 26	9	8	8
**12**	V1A and V1B	M, 30	9	9	8
**13**	V1A and V1B	M, 63	9	9	9
**14**	V1A and V1B	F, 69	10	9	10
		**Mean**	8.57	8.43	8.21
		**SD**	0.94	1.28	1.25

As pilot-testing results were positive, no further changes were made to the videos.

 Additional file 1: Appendix 1 displays the final scripts side-by-side to clearly demonstrate differences between them. Copies of the videos can be supplied on request.

### Stage 5: Administering the videos

#### Choosing viewers

Previous vignette studies examining health communication have used healthy volunteers as analogue patients [14, 50–54], and evidence suggests acceptable external validity of using healthy volunteers as APs [36]. One study demonstrated no difference in engagement with vignettes between disease-naïve and actual patients after age-matching, suggesting no difference in ecological validity for studies using disease-naïve volunteers vs patients [55].

Accordingly, we chose to recruit ‘healthy’ volunteers (that is, members of the general public as opposed to people from specific patient groups) as APs. Moreover, recruiting sufficient participants to ensure that the study was adequately powered was important, and we felt this more achievable with healthy volunteers. We aimed to recruit a diverse sample of participants regarding age, gender and ethnicity: anyone aged 18 or over, currently living in the UK was eligible. Participants were not compensated for their participation. We excluded medical doctors/students because we felt their medical knowledge and experience might influence results and produce conclusions less generalisable to the wider population.

#### Online setting

We administered the videos using Thiscovery, an online platform developed by The Healthcare Improvement Studies (THIS) institute. Participants watched the videos and completed questionnaires on their own electronic devices, without researcher supervision. To mitigate against external distractions and influences, we instructed participants to watch the videos on their own, at a time unlikely to be disturbed. Although participants could access the study on mobile phone devices, we advised them to use a larger screen if possible (ideally a computer/laptop or tablet) to make the experience more immersive.

#### Number of videos per viewer

Previous studies have varied in the number of vignettes watched per participant: from one [13, 14, 56, 57], to two [50, 58, 59], to four [47].

Our study was a randomised crossover trial, in which participants sequentially watched either V1A and V1B, or V2A and V2B (Fig. 2). As all our participants watched two videos, they were able to directly compare them and indicate a preference in communication style. Each participant acted as their own control, increasing power: crossover trials require lower sample sizes than parallel-group trials to meet the same criteria in terms of type I and type II error risks [60–62]. It is, however, important to acknowledge that within subjects designs may artificially inflate effects: in real healthcare settings, patients are very unlikely to experience two similar consultations in such a way.

Carryover effects – when the effect of the first treatment continues until the next period and alters the effect of the next treatment – can be a problem for crossover trials [61]. In our study, there was a risk that watching the first video may prime participants to think differently about the second video. This is a problem if the carryover effect from watching video A first differs from the carryover effect from watching video B first. Here, watching the high communication video first might prime participants to focus on uncertainty more closely than watching the low communication video first. 

To combat carryover effects, crossover studies often include ‘washout periods’. Some vignette studies have used these (e.g. a distraction task involving looking at an aquarium while listening to classical music) [47]. Despite limited evidence to suggest that such distraction tasks are effective in reducing carryover, we included one given the potential benefit and lack of obvious harms (even if ineffective). We designed a task which did not involve mental arithmetic so as not to bias against certain groups (e.g. less educated or numerically confident). Between watching videos, participants were presented with three pairs of photographs, and were asked to choose their preferred i. place for a picnic, ii. place for a walk, and iii. place to enjoy the view. 

#### Informing/debriefing participants

To avoid providing participants with cues which might change how they respond, participants were blinded to the study hypotheses. In the participant information sheet the aims of the study were described as examining the effects that different types of doctor communication might have on patients. We did not mention the concept of ‘diagnostic uncertainty’ prior to participation. After watching both videos and completing the questionnaires, we provided participants with a debriefing statement explaining the study aims and hypotheses.

## Discussion

We have outlined our application of published guidelines to produce four video vignettes used to study the communication of diagnostic uncertainty. We wish to emphasise two aspects of our methodology: firstly, the creation of high-quality vignettes in a cost-effective manner and, secondly, the use of an online platform. We argue that these points are particularly important in making video vignette methodology more accessible – both to researchers, and to diverse patient populations. Below, we draw from our experience to make recommendations for researchers designing video vignette studies.

### A pragmatic approach to producing videos

The creation of video vignettes can be time-consuming and costly, particularly if filming involves professional actors and/or film crews. These costs can quickly multiply if reshooting is required following pilot testing. Thus, although video vignettes are a useful tool in studying healthcare communication, they may appear inaccessible to researchers with limited resources.

This paper demonstrates that producing and using video vignettes need not necessarily be expensive and time-consuming. We filmed our videos with readily available and user-friendly technology, and we pursued a ‘video consultation’ style; this eliminated the need for extensive technical knowledge or equipment. We also carefully developed scripts and took a pragmatic approach to pilot-testing, saving both pilot testers’ and researchers’ time.

#### Approach to script development and pilot-testing

Existing vignette studies have taken a variety of approaches to pilot-testing: some have only pilot-tested scripts, others have tested scripts and videos, and some have not reported any formal pilot-testing [3]. There is wide variation in the number of pilot participants – ranging from ten in one study [44], to 116 laypeople and 46 cancer patients in another [19]. Notably, published guidelines on video vignette methodology do not state how extensive pilot-testing needs to be, and little research has specifically addressed this question.

Recruitment can be challenging in health communication research, and pilot-testing with large numbers may not be feasible. Furthermore, pilot-testing with more participants than is necessary raises ethical issues – we should avoid using participants’ time unless their involvement will positively impact the study.

We achieved good results with relatively small pilot numbers: 15 script pilot-testers and 14 video pilot-testers. The final videos were internally valid (the manipulations in uncertainty communication were perceived by participants as intended by the research team), and externally valid (they were rated as realistic and participants reported that they were able to adequately imagine themselves in the position of the patients).

These positive pilot-testing results may reflect the fact that we took steps at an early stage in the design process to ensure validity. Other studies have taken various approaches to initial script development: some have based them upon real consultations (recordings [11, 63] or direct real-time observations [22]), while others have used experts’ input [19]. A strength of our approach was the use of data from CODUS 1 to develop the scripts. This – combined with the use of expert input – helped us to create initial scripts that were reflective of real patient-doctor communication.

While the optimal number of pilot-testers is unclear, it is likely that the more steps taken early in the development of vignettes, the less extensive the pilot-testing needs to be. Our results suggest that if care is taken early in the development of the vignette scripts to maximise ecological validity, it is possible to produce high quality vignettes with relatively modest pilot-testing. This approach has dual cost and time-saving potential: first, researchers do not need to recruit excessive numbers of pilot testers; second, resulting videos are more likely to be valid, therefore reducing the likelihood of needing to re-shoot.

#### Filming considerations

Existing studies have used professional film crews, actors and script-writing experts, with some filming over several days with multiple cameras to produce the videos [22, 23, 64]. Such extensive processes, although commendable, may not be accessible for researchers with smaller budgets or less time. Importantly, our results show that they are not necessary to produce valid vignettes.

We filmed our vignettes using a single camera angle on an iPhone; we did not employ any professional camera crew. Our pilot-testing demonstrates that good quality videos can be produced with relatively minimal equipment and without input from filming experts. Moreover, APs are increasingly familiar with video consultations, so less formal, single (face-on) camera angle videos may be more realistic.

Although it may have been more naturalistic for the doctor to have memorised the text (as opposed to reading from the teleprompter), we had limited time and we wanted to ensure that there was no deviation from the scripts we had already pilot-tested. We were unable to find any published data comparing the realism of memorisation vs reading from a teleprompter, but this is something future researchers could consider if time permits reliable memorisation of the scripts.

Despite suggestions that an actor might be more realistic, we found otherwise. Costs may be saved by using volunteers (for example, from the research team or their contacts) in place of professional actors. Alternatively, researchers who decide to use an actor might find it beneficial to perform a screen test with preliminary pilot-testing initially, before employing them for the entire series, to save unnecessary costs.

### Accessibility and diversity considerations.

Online vignette studies may increase accessibility for participants, but current research largely overlooks accessibility considerations. With increasing use of online methods [65], it is unsurprising that many studies have delivered video vignettes online [11, 20, 56, 66]. However, many vignette studies fail to report the study setting (the viewing location and its arrangement – for example, whether participants participated entirely online by watching vignettes on their own devices, or whether they attended an in-person viewing). Of those that do, few justify these choices [3], and even fewer critically reflect on their inclusion or accessibility implications [67]. This reflects the lack of research considering how online video vignette studies may be more or less accessible to participants from different groups. 

Below we evaluate the little existing research on accessibility and online methods, and urge researchers to consider how they might account for the needs of underrepresented groups (for example, those with hearing or visual impairment) in vignette studies. For instance, working with stakeholder groups to co-design different conditions related to accessibility, and utilising online platforms to recruit greater sample sizes, may increase inclusivity [67]. 

#### Diversity considerations in online recruitment and vignette delivery

Although online technologies can widen participation in research, they can also create barriers to participation by favouring those with good digital literacy and access [65, 68, 69]. Similarly, although social media recruitment enrolment may be effective, consideration of representation is needed: evidence suggests that social media recruitment might yield a less demographically diverse sample [70]. It is notable that most literature focuses on research methods like online surveys or interviews [69, 71]; critical conversations around the ethics of online vignette studies are needed. 

#### Video vignettes and accessibility 

When creating our videos we consulted the literature on accessibility. Our choices reflect attempts to balance varying participant needs with logistical constraints the need to ensure validity in results.

For video vignette studies, alternative forms of consuming video content – such as subtitles or closed captions, or providing a written alternative which can be read in Braille or screen-readers – could make the research more inclusive [68]. One study in Belgium reported use of subtitles [20], and another recommended piloting with community stakeholders familiar with accessibility concerns [67]. There is, however, some concern that changing the modality of vignettes may change how participants interact with them, making interpretation of results challenging. For example, in our study, enabling a rewind/replay function and subtitles would have allowed those who are hard of hearing to better engage with the content. We did not do this, due to concerns that watching the video multiple times (something which cannot be done in real consultations), or reading the words on screen might alter the interpretation of the content of the consultation. Such potential differences would pose difficulties for comparing results between those who listened to the consultation and those who read captions.

Such concerns are not, however, evidence-based. A recent study investigating the impact of vignette modality (written, audio and video) showed no effect on engagement, recall, trust, satisfaction and anxiety [40]; this suggests that changing the modality of vignettes depending on participant need (e.g., adding subtitles) may not have any detrimental impact on research validity. In fact, data quality may actually be enhanced if researchers can design for inclusion by providing alternative forms of the same vignette without compromising validity, as wider groups may be included [68]. Nonetheless, further research is needed to critically explore these issues.

Overall, there is limited conclusive guidance on how to reduce barriers to participation in vignette studies for those from underserved groups – those from minority backgrounds or who have disabilities like dyslexia, visual/hearing impairment and learning disabilities [67]. Further research into how inclusive research design (specifically, exploration of whether alterations to the mode of the vignette influences outcomes) might influence results is needed. 

## Conclusion

Four video vignettes manipulating the communication of diagnostic uncertainty were created and validated for experimental use. Our reflections provide practically useful recommendations of how to make video vignettes more accessible both to researchers and to participants from a range of backgrounds.

We propose that it is possible to produce high quality vignettes without an overly complex or expensive development procedure, potentially increasing accessibility for researchers with budget/time constraints. We highlight the potential benefits of online methods in improving accessibility for participants but suggest a need to acknowledge and explore how to reduce the barriers to participation in online vignette studies for those from underserved groups.

### Supplementary Information

**Additional file 1: Appendix 1.** Vignette scripts and introductory text.

## Data Availability

Copies of the scripts developed in this study can be found in Additional file 1: Appendix 1. Copies of the final video vignettes produced can be provided on request (please contact the corresponding author).

## Footnotes

1 The findings of the main study are being prepared for publication in a separate manuscript. This paper is intended to detail the development of the vignettes and the decisions around the study design, rather than report the results of the main study itself.

2 In a separate study, we have also been exploring real consultations to gain a greater understanding of the whole diagnostic process; our observations in three UK hospitals corroborate our CODUS 1 findings that there is significant variation in the way diagnostic uncertainty is communicated.

3 We did not formally assess or measure non-verbal behaviour as part of our pilot-testing, instead relying on global assessments by the author team (the authors reviewing different takes, and selecting those where they felt the non-verbal communication was similar across vignettes). As part of the review process, both anonymous reviewers suggested that we could have shown muted versions of the vignettes to naive pilot-testers, for example using a nonverbal immediacy scale and comparing it across conditions to ensure there were no significant differences in nonverbal behaviours. We thank them for this suggestion, and although this is not an approach we used in this study, we would encourage researchers to consider it in future vignette studies.
