# Supplementary material for: Creating and administering video vignettes for a study examining the communication of diagnostic uncertainty: methodological insights to improve accessibility for researchers and participants
Source: BMC Med Res Methodol. 2023 Dec 15;23:296. doi: 10.1186/s12874-023-02072-7 (PMC10722843; doi:10.1186/s12874-023-02072-7)
Supplement: Supplementary file 1 — Additional file 1: Appendix 1. Vignette scripts and introductory text. [file 12874_2023_2072_MOESM1_ESM.docx]

# Appendix 1: vignette scripts and introductory text

# Scenario 1: Headache

## 1.1 Introductory text to be provided to participants

Please read the following scenario, and try to imagine yourself in the place of the patient.

You are in A&E, having come in a few hours ago with a headache. The headache came on earlier today when you were sitting down. It took a few minutes to reach its worst intensity. It was the worst headache you’ve ever had – the pain was very severe and affected your whole head. You have never had this sort of headache before.

You haven’t noticed any other symptoms – no neck pain, no rashes, no change to your vision, no fainting/blacking out and no sickness or vomiting. You have not had a fever.

You were concerned by the severity of the headache, so you decided to attend A&E. You were seen by a doctor, who asked about your symptoms and then examined you. They organised for you to have a number of tests – blood tests and a CT scan of your head.

You have now been in A&E for several hours. You have been given some paracetamol and the headache has much improved – it is now a dull ache, about 3/10 severity.

You are now in a cubicle waiting to see the doctor again. The doctor is back to tell you the results of the investigations and explain what is going to happen next.

## 1.2 V1A script (high communication of uncertainty)

Hello again, I’m Dr Jones – we met a few hours ago when you first came in. The nurse was just telling me that you’re feeling a bit better, which is great news.

As you know, we’ve done a thorough examination and some tests, and the bottom line is that they are all normal. But let me explain why we've done those. Whenever someone comes into hospital with a really severe headache like you had, we always need to think about whether something serious or life-threatening might be causing it. Of course, most headaches aren’t anything to worry about, but a small number can be caused by something serious.

The sort of things we want to rule out are meningitis, brain tumours, And perhaps the most important thing to rule out in this kind of situation is the possibility of a bleed in the brain, which is called a subarachnoid haemorrhage. Based on the blood tests and CT scan, I am very confident that you don’t have meningitis or a brain tumour. The CT scan also looks for any evidence of bleeding in the brain, and in your case there isn’t any - as I said, the scan is normal.

However, the scan is not 100% reliable at ruling out bleeds. So in some cases we don’t see anything abnormal on the scan, but there is still a very small bleed. In some situations we go on to do a further test, called a lumbar puncture, to more accurately rule out whether there has been a bleed. This involves putting a needle into the back to collect some fluid from around the spinal cord.

The good news is that in your case, we did the scan quite soon after the headache started which makes it more sensitive at picking up bleeds. This means that, although I can’t say with 100% certainty that you haven’t had a bleed, the risk is incredibly low.

Because of all this, I don’t think we need to do that extra test, the lumbar puncture. The fact that your pain is so much better now, your examination was normal, and we have a normal CT scan, this is all very reassuring. And these lumbar punctures do carry risks in themselves. In your case, the probability of there being a bleed is so small that doing a lumbar puncture would carry more risk than benefit.

It's difficult for us to know exactly what caused this headache – it could perhaps be a migraine, but I don’t know for sure. The main thing is that at this point there’s no evidence of anything serious or life-threatening, and I don't think any further investigations are needed, so I think you are safe to go home. We can give you some more painkillers, and I would expect that the headache will get better.

Of course, as I say, we can never be completely certain about these things. If you feel like your pain is getting worse, or if you develop any new symptoms, such as arm or leg weakness, or blurred vision, or anything else is worrying you, please come back to hospital, as we would need to revisit the whole situation. In particular, we might think about doing that extra test, the lumbar puncture.

Does that all make sense? Do have questions?

## 1.3 V1B script (Low communication of uncertainty)

Hello again, I’m Dr Jones – we met a few hours ago when you first came in. The nurse was just telling me that you’re feeling a bit better, which is great news.

As you know, we’ve done a thorough examination and some tests, and the bottom line is that they are all normal. But let me explain why we've done those. Whenever someone comes into hospital with a really severe headache like you had, we always need to think about whether something serious or life-threatening might be causing it. Of course, most headaches aren’t anything to worry about, but a small number can be caused by something serious.

But as I say, your test results are very reassuring.

Your blood tests are all normal, and there’s nothing to suggest in your examination that there’s anything else causing the headache at the moment. The CT scan also didn’t show any evidence of bleeding within the brain, something called a subarachnoid haemorrage which is one of the things that we think about in these severe headaches.

The fact that all the tests were reported as normal is very reassuring for us. At this point there’s no evidence of anything serious or life-threatening, and I don't think any further investigations are needed, so I think you are safe to go home. We can give you some more painkillers, and I would expect that the headache will get better.

Of course, if you feel like your pain is getting worse, or if you develop any other symptoms, such as arm or leg weakness or blurred vision. or anything else is worrying you, please come back to the hospital.

Does that all make sense? Do have questions?

## 1.4 Comparison of scripts for scenario 1 (headache)

| Script component | High DU script | Low DU script |
| --- | --- | --- |
| Introduction | Hi, I’m Dr Jones – we met a few hours ago when you first came in. The nurse has just been telling me that you’re feeling a bit better, which is great. | Hi, I’m Dr Jones – we met a few hours ago when you first came in. The nurse has just been telling me that you’re feeling a bit better, which is great. |
| Explanation of normal results and rationale for doing tests | As you know, we’ve done a thorough examination and a-few tests, and the bottom line is that they are all normal.  But I’d like just take a minute to explain why we’ve done the tests and what that the results mean.  While most headaches are nothing to worry about, a small number can be serious, and we obviously try to rule out these serious causes.  The sort of things we want to rule out are meningitis, brain tumours, and perhaps the most important thing to rule out in the type of headache you’ve had, is the possibility of a bleed on the brain, which we call a subarachnoid haemorrhage.  So, based on the blood tests and CT scan, I am very confident you do not have meningitis or a brain tumour. The CT scan, also does not show any evidence of bleeding on the brain. . | As you know, we’ve done a thorough examination and a-few tests, and the bottom line is that they are all normal.  But I’d like to just take a minute to explain why we’ve done the tests and what that the results mean.  While most headaches are nothing to worry about, a small number can be serious, and we obviously try to rule out these serious causes.  The CT scan didn’t show any evidence of bleeding within the brain, something called a subarachnoid haemorrhage, which is one of the things that we think about when someone comes in with a severe headache. Fortunatley, Your blood tests are also all normal, and there’s nothing to suggest in your examination that there’s anything untoward causing the headache at the moment. Overall, then |
| Discussion about uncertainty surrounding the investigation results and possibility of LP | However, in some cases we don’t see anything abnormal on the scan, but there is still a very small bleed. Sometimes we do a further test, called a lumbar puncture, to more accurately rule out whether there’s been a bleed or not. This involves putting a needle into the back to collect some fluid from around the spinal cord.  The good news is that in your case, we did the scan quite soon after the headache began, which makes it more sensitive at picking up bleeds. And this means that, although I can’t say with 100% certainty that you haven’t had a bleed, the risk is incredibly low.  Because of all this, I don’t think we need to do that extra (lumbar puncture) test.  So, the fact that your pain is much better now, your examination was normal, and we have a normal CT scan, means I’m very reassured. The extra lumbar puncture test does carry a risk in itself , so in your case, the probability of there being a bleed is so small that doing a lumbar puncture would carry more risk than benefit. |  |
| Acknowledgement of ongoing uncertainty as to cause of symptoms | So, It's difficult for us to know exactly what caused the headache – it could perhaps be a migraine, but I don’t know for sure. The main thing is that at this point, |  |
| Reassurance | There’s no evidence of anything serious or life-threatening, and I don't think any further investigations are needed.  So I think you are safe to go home. We can give you some more painkillers, and I would expect that the headache will get better. | there’s no evidence of anything serious or life-threatening, and I don't think any further investigations are needed.  So I think you are safe to go home. We can give you some more painkillers, and I would expect that the headache will get better. |
| Safety-netting | Of course, as I say, we can never be completely certain about these things. If you do feel like your pain is getting worse, or if you do develop any new symptoms, such as arm or leg weakness, or blurred vision, or anything else that’s worrying you, please come back to hospital, and we can re-evaluation the situation, and we can perhaps consider doing that extra lumbar puncture test.  Does that all make sense? Do have questions?  Ok  Thank you | Of course, if you feel like your pain is getting worse, or if you develop any new symptoms, such as arm or leg weakness or blurred vision, or anything else is worrying you, please come back to the hospital.  Does that all make sense? Do have questions?  Ok  Thank you |

## 2. Scenario 2: change in bowel habit

## 2.1 Introductory text be provided to participants

Please read the following scenario, and try to imagine yourself in the place of the patient.

You have been suffering from vague problems with your gut on and off for the last 3 years. You intermittently have mild lower abdominal pain (which is relieved when you go to the toilet), and bloating. You also have periods of time when you are constipated, and other periods when you have loose stools.

You are otherwise quite well. You haven’t lost any weight, you have never noticed any blood in your stools, and you have not had any nausea or vomiting.

You first went to the doctor a few weeks ago to get your symptoms checked out. The doctor listened to your story, examined you and organised for you to have a number of tests (blood tests and a stool sample).

You are now going back to the doctor to get the results of the investigations.

## 2.2. V2A script (High communication of uncertainty)

Hello, I’m Dr Jones – we met a few weeks ago when your first came to see me. Thank-you for coming back in today. You told me last time about the symptoms you’ve been having over the last few years. I’m sorry they’ve been causing you problems; you did the right thing by coming in to get things checked out.

As part of that we’ve done quite a few tests. We’ve now got the results back, and the good news is they’re all completely normal.

Let me explain why we were doing those tests. Some were looking at your blood count, your liver and kidney function, your thyroid function – they are all normal. We also did some more specific tests looking for other conditions which can cause problems with the gut, such as coeliac disease or problems with the pancreas. Importantly, we also did a test which checks for any blood in the stool, which was negative.

In situations like this, we always want to make sure we aren’t missing a sinister or life-threatening cause for the symptoms. In your case, these would be conditions such as inflammatory bowel disease – for example Crohn’s disease, which you might have heard of – or even something like a bowel cancer. Based on your test results, as well as the symptoms you’ve been having, it’s unlikely that this is inflammatory bowel disease. I also think it’s very unlikely to be cancer – you don’t have any blood in your stools and you don’t have any what we call ‘red flag symptoms’. You’ve also had the symptoms for three years, and with an untreated bowel cancer, I would expect it to progress and possibly cause more trouble in that time, which it hasn’t done.

So, all of your tests have been reassuring, but they haven’t shown a clear definitive cause for your symptoms.

Taking everything into account, the most likely diagnosis here is something called irritable bowel syndrome, or IBS. It’s what’s referred to as a functional condition. And by that we mean the gut just isn’t functioning quite normally. It’s not a disorder we completely understand, but essentially the gut is oversensitive, and people end up with symptoms like you’ve been getting.

But there are certain things that we can do to help manage the condition – it’s things like diet changes and some medicines to control the symptoms. I can give you some information on these and moving forward we can make a plan.

In summary, although there is no specific test for IBS, I think it’s probably what’s going on here. There are no worrying features to suggest a more dangerous or sinister condition.

Of course, if anything changes or your symptoms get worse – for example, if you notice any blood in your stools or you start losing weight – you should come back and see us. We would then need to think about reinvestigating to make sure we haven’t missed anything, as there are some other tests that we could do. They’d include us doing a camera test to look directly at the bowel or perhaps a CT scan. At the moment I don’t think these tests are needed, but if anything changes, we would need to revisit the whole situation.

How does that all sound? Do you have questions?

## 2.3 V2B script (Low communication of uncertainty)

Hello, I’m Dr Jones – we met a few weeks ago when your first came to see me. Thank-you for coming back in today. You told me last time about the symptoms you’ve been having over the last few years. I’m sorry they’ve been causing you problems; you did the right thing by coming in to get things checked out.

As part of that we’ve done quite a few tests. We’ve now got all of your results back, and the good news is they’re all completely normal.

We’ve done blood and stool tests, and importantly, we also did a test which checks for any blood in the stool, which was negative.

In situations like this, we always want to make sure we aren’t missing a sinister or life-threatening cause for the symptoms. All of your tests have been reassuring. These tests, along with the normal examination findings, have ruled out any sinister or life-threatening causes for the symptoms.

Taking everything into account, the diagnosis I’ve come to for your symptoms is irritable bowel syndrome, or IBS. It’s what’s referred to as a functional condition. And by that we mean the gut just isn’t functioning quite normally. Essentially the gut is oversensitive, and people end up with symptoms like you’ve been getting.

But there are certain things that we can do to help manage the condition – it’s things like diet changes and some medicines to control the symptoms. I can give you some information on these and we can make a plan.

So in summary, your symptoms are due to a common condition called Irritable Bowel Syndrome. The normal investigations we have ruled out the more dangerous or sinister conditions so we don’t need to do any more invasive tests at the moment.

Of course, if anything changes or your symptoms get worse – for example, if you notice any blood in your stools or you start losing weight – you should come back and see us.

How does that all sound? Do you have questions?

## 2.4 Comparison of scripts for scenario 2 (change in bowel habit)

| Script component | High DU script | Low DU script |
| --- | --- | --- |
| Introduction | Hi, I’m Dr Jones – we met a few weeks ago when your first came to see me. Thank you for coming back in today. You told me last time about the symptoms you’ve been having over the last few years. I’m sorry they’ve been causing you problems; you absolutely did the right thing by coming in to get things checked out. | Hi I’m Dr Jones – we met a few weeks ago when your first came to see me. Thank you for coming back in today. You told me last time about the symptoms you’ve been having over the last few years. I’m sorry they’ve been causing you problems; you absolutely did the right thing by coming in to get things checked out. |
| Explanation of Ix and ddx | As you know, we did quite a few tests. We’ve now got all of your results back, and the good news is they’re all completely normal.  Let me explain why we were doing those tests. Some of them were standard: we checked your full blood count, and your liver and kidney and thyroid tests, and they were all normal.  In situations like this, we always want to make sure we aren’t missing a sinister or life-threatening cause for the symptoms. In your case, these would be conditions such as inflammatory bowel disease – for example Crohn’s disease, which you might have heard of – or something like a bowel cancer. Based on your blood test results, as well as the symptoms you’ve been having, it’s unlikely that this is inflammatory bowel disease. I also think it’s very unlikely to be cancer – you don’t have any blood in your stools and you don’t have any what we call ‘red flag symptoms’ which would make us worry about cancer. In addition, you had the symptoms for three years, and with an untreated bowel cancer, I would expect it to progress and possibly cause more trouble in that time, which it hasn’t done.  So, all of your tests have been reassuring, but they haven’t shown a clear definitive cause for your symptoms. | As you know, we did quite a few tests. We’ve now got all of your results back, and the good news is they’re all completely normal.  Let me explain why we were doing those tests. Some of them were standard: we checked your full blood count, and your liver and kidney and thyroid tests, and they were all normal.  In situations like this, we always want to make sure we aren’t missing a sinister or life-threatening cause for the symptoms.  So we also did some more specialised blood tests, and they were normal too. Importantly, we also did a test which checks for any blood in the stool, which was negative. |
| Explanation about IBS | Taking everything into account, the most likely diagnosis here is something called irritable bowel syndrome, or IBS. It’s what’s referred to as a functional condition. And by that we mean the gut just isn’t functioning quite normally. It’s not a disorder we completely understand, but essentially the gut is oversensitive, and people end up with symptoms like you’ve been getting.  But there are certain things that we can do to help manage the condition – things like dietary changes and there are some medicines which help control the symptoms. I can give you some information on these and moving forward we can make a plan. | Taking everything into account, the diagnosis I’ve come to for your symptoms is irritable bowel syndrome, or IBS. It’s what’s referred to as a functional condition. And by that we mean the gut just isn’t functioning quite normally. Essentially the gut is oversensitive, and people end up with symptoms like you’ve been getting.  But there are certain things that we can do to help manage the condition – things like dietary changes and there are some medicines which help control the symptoms. I can give you some information on these and we can make a plan. |
| Reassurance | So in summary, while there is no specific test for IBS, I think it’s probably what’s going on here. There are no worrying features at present to suggest a more dangerous or sinister condition, such as inflammatory bowel disease or bowel cancer. | So in summary, your symptoms are due to a common condition called Irritable Bowel Syndrome. The normal investigations we have ruled out the more dangerous or sinister conditions so we don’t need to do any more invasive tests at the moment. |
| Safety-netting | Of course, if anything changes or your symptoms get worse – for example, if you notice any blood in your stools or you start losing weight – you should come back and see us. We would then need to think about reinvestigating to make sure we haven’t missed anything, as there are some other tests that we could do. They’d include us doing a camera test to look directly at the bowel or perhaps a CT scan. At the moment I don’t these tests are needed, but if anything changes, we would need to revisit the whole situation.  How does that all sound? Do you have questions?  OK, thank you | Of course, if anything changes or your symptoms get worse – for example, if you notice any blood in your stools or you start losing weight – you should come back and see us.  How does that all sound? Do you have questions?  OK, thank you |
